# The Reliability, Validity, and Normative Data of Interpupillary Distance and Pupil Diameter Using Eye-Tracking Technology

**DOI:** 10.1167/tvst.6.4.2

**Published:** 2017-07-03

**Authors:** Nicholas P. Murray, Melissa Hunfalvay, Takumi Bolte

**Affiliations:** 1 East Carolina University, Greenville, NC, USA; 2 Science Department, RightEye, LLC, Bethesda, MD, USA; 3 Clemson University, Clemson, SC, USA

**Keywords:** reliability, validity, normative data, interpupillary distance, pupil diameter

## Abstract

**Purpose:**

The purpose of this study was to determine the reliability of interpupillary distance (IPD) and pupil diameter (PD) measures using an infrared eye tracker and central point stimuli. Validity of the test compared to known clinical tools was determined, and normative data was established against which individuals can measure themselves.

**Methods:**

Participants (416) across various demographics were examined for normative data. Of these, 50 were examined for reliability and validity. Validity for IPD measured the test (RightEye IPD/PD) against the PL850 Pupilometer and the Essilor Digital CRP. For PD, the test was measured against the Rosenbaum Pocket Vision Screener (RPVS). Reliability was analyzed with intraclass correlation coefficients (ICC) between trials with Cronbach's alpha (CA) and the standard error of measurement for each ICC. Convergent validity was investigated by calculating the bivariate correlation coefficient.

**Results:**

Reliability results were strong (CA > 0.7) for all measures. High positive significant correlations were found between the RightEye IPD test and the PL850 Pupilometer (*P* < 0.001) and Essilor Digital CRP (*P* < 0.001) and for the RightEye PD test and the RPVS (*P* < 0.001).

**Conclusions:**

Using infrared eye tracking and the RightEye IPD/PD test stimuli, reliable and accurate measures of IPD and PD were found. Results from normative data showed an adequate comparison for people with normal vision development.

**Translational Relevance:**

Results revealed a central point of fixation may remove variability in examining PD reliably using infrared eye tracking when consistent environmental and experimental procedures are conducted.

## Introduction

Distance between the pupils, called interpupillary distance (IPD), is an important clinical measure used to identify potential vision issues such as stereo acuity,^1^ near point convergence,^2^ accommodation,^3^ and other vision-related issues.^4^ Furthermore, normative IPD data is important in the optical industry when fitting patients for glasses. IPD is measured using the distance between the centers of the pupils.^5,6^

Diameter of the pupil (PD) is another component of the eye that is measured and is related to image quality. A larger pupil will allow more peripheral rays into the eye, resulting in high-order monochromatic aberrations that pose a problem with image quality when the PD is large.^7^ A limitation of very small pupils can be diffraction; however, this problem is less significant than the aberrations in larger pupil sizes as demonstrated by Howland and Howland.^8^ Depth of focus is related to pupil size. Smaller pupils allow an increase in depth of focus, which in turn reduces the effect of refractive errors and errors in accommodation such as accommodative lag on the quality (blur) of the retinal image.^9^

Interpupillary distance and PD influence many vision components that are important in activities of daily living (ADLs) as well as peak performance in athletes and fighter pilots. For instance, IPD determines the amount of stereo separation of two images that are combined in the brain to produce stereo perception.^10,11^ Stereo perception is important in rapid three-dimensional processing involved in driving a vehicle and catching a ball. It is therefore important to be able to measure these metrics reliability and accurately and to determine normative data against which a person can be measured.

Numerous methods have been used in the measurement of IPD; Viktorins method, pupilometers, and corneal reflection are each commonly used in clinical settings. Viktorins method uses a hand measure of IPD by measuring the distance between certain features in the eye. Pupilometers are handheld ophthalmological devices that measure distance between the pupils by aligning the device with the corneal reflexes of the participant. Corneal reflection, the method used in the present study, measures the distance between the pupils through the reflection of infrared light on the eyes.

Various inaccuracies in these measurement processes have been discussed, including parallax error (a large difference between the IPD of observer and participant), incorrect spacing between the participant and the observer, and/or incorrect positioning of measurement tools.^5^ Obstfeld and Chou^12^ examined nine of the leading pupilometers and found an average of 2.3-mm error (SD = 0.26 mm). Sources of error were poor eye relief in all pupilometers and friction in the scale adjustments. However, the pupilometers were found to give consistent readings (i.e., were reliable) within the limits of clinically acceptable parameters. However, the accuracy (i.e., the validity) was questioned, especially in IPDs that were especially large or small. Viktorins method and pupilometer measurements have been criticized for lack of examination of reliability, and in the few studies where reliability has been examined, results have revealed poor reliability.^5^ One possible reason for low reliability may not reside in the instruments themselves but in the technician's difficulty using such instruments.^13^

Numerous methods have been used in the measurement of PD, such as lens systems with millimeter scales (e.g., Colvard; Oasis Medical, Glendora, CA); dynamic and binocular PD measurements using infrared light (e.g., P2000SA; Procyon Instruments, London, UK); and wavelength aberrometers based on the Hartmann-Shack principle (e.g., WASCA; Asclepion-Meditec-Zeiss, Jena, Germany). In a study by Schmitz et al.,^14^ pairwise comparison between the lens systems, infrared light, and wavelength aberrometers showed statistically significant (*P* < 0.05) differences in median deviations. Authors argue, however, that these differences (ranging up to 1 mm) were not clinically significant.

The form of comparison method employed by the Rosenbaum Pocket Vision Screener (RPVS) was first described in 1863 by Follin.^15^ It is still a widely used method of measuring pupil size in clinical practice.^16^ It is a low-cost alternative to the lens systems, infrared systems, and aberrometers. The RPVS is a commonly used tool to measure pupil diameter (PD) for nonsurgical patients.^17^

Pupil diameter has also been measured using infrared eye trackers^18^ (e.g., Tobii X120, Tobii T120; Tobii Technology, Stockholm, Sweden, and Eye Link 1000; SR Research, Kanata, Canada). Brisson et al.^18^ found that pupil size can be over- and underestimated in infrared eye trackers depending on gaze position. When the eyes are positioned straight ahead, the pupil is most accurately measured, ranging from 0.3 to 1.0 mm, depending on the eye tracker, with the Eye Link 1000 producing the best results. When the eyes look away from the center, systematic errors occur in measuring PD as the pupil appears squashed. When looking straight ahead, the pupil appears circular, therefore providing the most accurate PD readings. When using a chin–forehead rest, results were even more accurate (<0.3 mm). Nevertheless, the reliability (repeatability) and validity (accuracy) is questionable and requires further examination of both the tool and the stimuli used to examine the PD readings before results can be considered with confidence.

Interpupillary distance and PD differ based on various demographic considerations. Dodgson^10^ used data from the Anthropometric Survey of United States Army Personnel database, consisting of 3976 participant, aged 17–51, and investigated population norms for IPDs. Dodgson suggested that although a mean IPD of the population was proposed as 63.36 mm, this value was inaccurate when analyzed for ethnicity, sex, and age. Significant differences were identified between ethnic groups for mean IPD, which was attributed to various physiological differences among ethnicities. Sex differences were also found, with males demonstrating significantly greater IPDs than females.^6,10^ Interpupillary distance has also been shown to change significantly with age until approximately 30 years of age, with most change occurring in the first 19 years of life.^19^ Given that IPD is influenced by ethnicity, sex, and age, it would appear prudent that these characteristics be identified within research to avoid inconsistent results due to potential confounding variables. Pupil diameter has also shown to vary based on age,^20,21^ sex,^22^ and ethnicity.^23^

The purpose of this study was to (1) determine the reliability of IPD and PD measures using an infrared eye tracker (120 Hz; TeHow, Germany) and central point stimuli (designed by RightEye, LLC, Bethesda, MD); (2) to examine the validity of the RightEye IPD Test compared to the PL850 Pupilometer (Hilco, Plainville, MA) and the Essilor Digital CRP (DAES03-001; Essilor, Charenton-le-Pont, France) for IPD, and the RPVS for PD measures (3908 Pocket Eye Chart; Medisave, Stratford, CT); (3) to establish normative data for which individuals can measure themselves.

## Methods

### Participants

Four hundred sixteen participants were selected for this study. Participants were recruited through advertisements placed on the internet, social media, bulletin boards, and via word of mouth. Of the group, 189 (45%) were males and 227 (55%) females. Participants were between the ages of 18 and 73 years (M = 42, SD = 8.7). Of the 416 participants, 68% were white, 17% black, 8% Hispanic, 1% Native American, and 6% opted not to report ethnicity.

To test reliability and validity, a small subgroup of 50 participants was tested a second time. The subgroup included participants between the ages of 24 and 73 years (M = 45, SD = 8.4); 48% were male (*n* = 24), and 52% were female (*n* = 26). Of the 50 participants, 70% were white, 15% black, 9% Hispanic, 0% Native American, and 6% opted not to report ethnicity.

Participants were excluded from participation in the study if they met any of the following prescreening conditions: neurological disorders (such as concussion, traumatic brain injury, Parkinson's disease, Huntington's disease, cerebral palsy); obvious vision-related issues that prevented successful calibration^24,25^ of all nine points (such as extreme tropias,^26^ phorias,^26,27^ static visual acuity of greater than 20/400,^24^ nystagmus,^24,28^ cataracts,^29^ or eyelash impediments^29^); small vessel strokes; or consumption of drugs or alcohol within 24 hours of testing. Participants removed any eyewear, including glasses or contact lenses. All participants provided informed consent to participate in this study in accordance with institutional review board procedure (IRB: UMCIRB 13-002660). This research followed the tenets of the Declaration of Helsinki.

Testing was conducted by vision specialists (e.g., optometrists, ophthalmologists) and, in the case of the RightEye IPD/PD test, the examiners had received and passed the RightEye training, education, and protocol procedures prior to testing.

### Materials and Equipment

**Figure 1:**
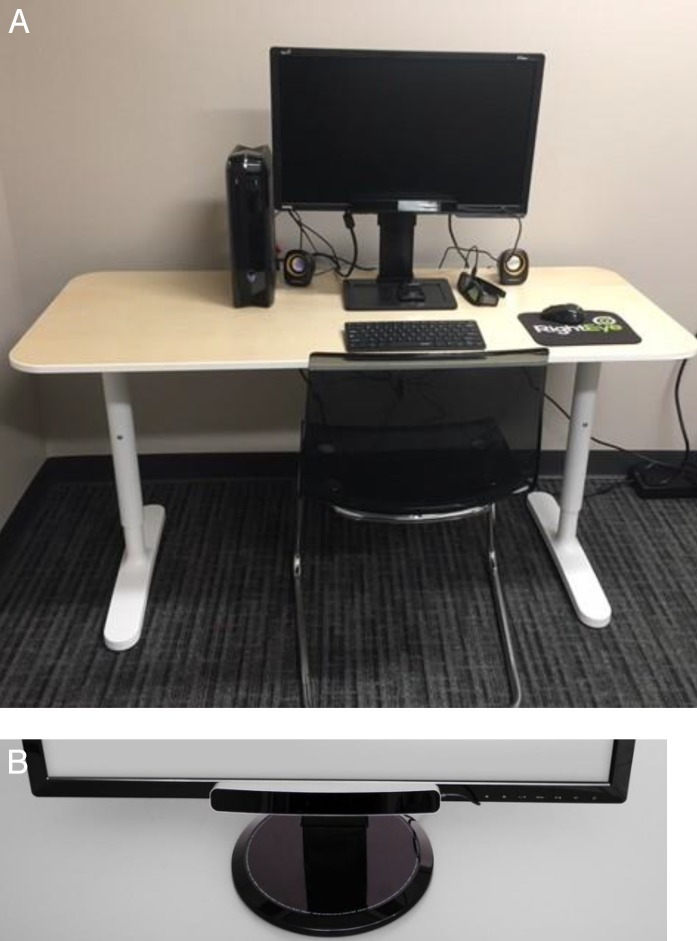
(A) RightEye set up. (B) Magnification of eye tracker.

For the RightEye IPD/PD test, the participants were seated in a stationary (nonwheeled) chair that could not be adjusted in height at a desk within a quiet, private testing room (see Fig. 1). The participants were asked to look at a NVIDIA 24-inch 3D Vision monitor (NViDiA, Santa Clara, CA) that could be adjusted in height and was fitted with an SMI 12” 120-Hz remote eye tracker connected to an Alienware gaming system (Miami, FL) and a Logitech (Y-R0017; Logitech, Romanel-sur-Morges, Switzerland) wireless keyboard and mouse. Screen luminance was 85 cd/m^2^ (229 lux), and room luminance with the lights on was 344 cd/m^2^ (102 lux). Participants' heads were unconstrained during the test, although they were instructed to sit still. The system has no restrictions in range when calculating IPD or PD.

The eye tracker is used to capture the x and y coordinates for each eye, along with the *z*-distance at 120 times a second. Once the stimulus is at the center point of the screen (960 × 540), the eye tracker detects if the eye is looking at the stimuli; once confirmed, the first sample of data is used to measure IPD. Then, using the x and y eye coordinates in three-dimensional space, participants' IPD is calculated for the left and right eyes.

Pupil diameter measurements are taken at the same time as IPD measurements in the RightEye IPD/PD test. For a 120-Hz eye tracker, output is reported every 8 milliseconds. Using the center point of the screen, 700 milliseconds of data are collected, resulting in a sample of 87 data points. These metrics are then used to calculate average pupil size, range, and standard deviation of both left and right eye. Size of the pupil is determined by the contour of the pupil.

#### PL850 Pupilometer

This digital pupilometer measures distance between the center of each pupil. Range is from 47 to 84 mm in 0.5mm steps.

#### Essilor Digital CRP

This corneal reflection pupilometer measures distance between the center of each pupil. Range is from 48 to 77 mm in 0.5-mm steps.

#### Rosenbaum Pocket Vision Screener

The RPVS is a pupil size chart (16 × 9 cm) that measures pupil size in millimeters ranging from 2 to 9 mm. Pupil measurements were made at 1.0-mm steps unless the pupil appeared between two circles on the card, and a 0.5-mm measurement interval was made.

### Testing Procedure

The RightEye IPD/PD test involved participants positioning themselves in front of the eye-tracking system, measured at an exact distance of 60 cm (ideal positioning within the head box range of the eye tracker) from the eye tracker for standardization before testing. A nine-point calibration test was conducted with points spanning the computer screen. Participants were required to pass all nine points before proceeding with testing.

**Figure 2:**
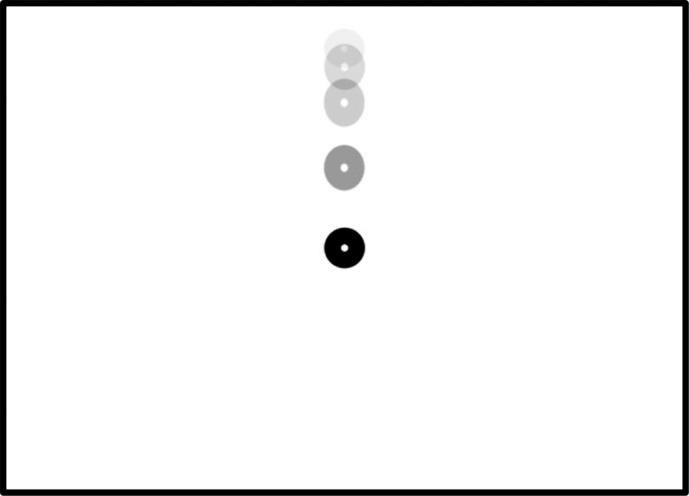
Stimuli.

Upon successful calibration, the RightEye IPD/PD test commenced. The participants read the following instructions: “Follow the dot from the top of the screen to the center. Watch the dot get smaller and keep looking at it until it disappears. Keep your eyes as still and focused when the dot stops in the center of the screen.” Binocular viewing conditions were used for testing, that is, data are collected simultaneously from the right and left eye. When instructions are read, the participant proceeds to the test where a dot drops from the top center of the screen to the middle of the screen (see Fig. 2). Once in the middle of the screen the dot stops and shrinks in size over a 700-millisecond period.

**Figure 3:**
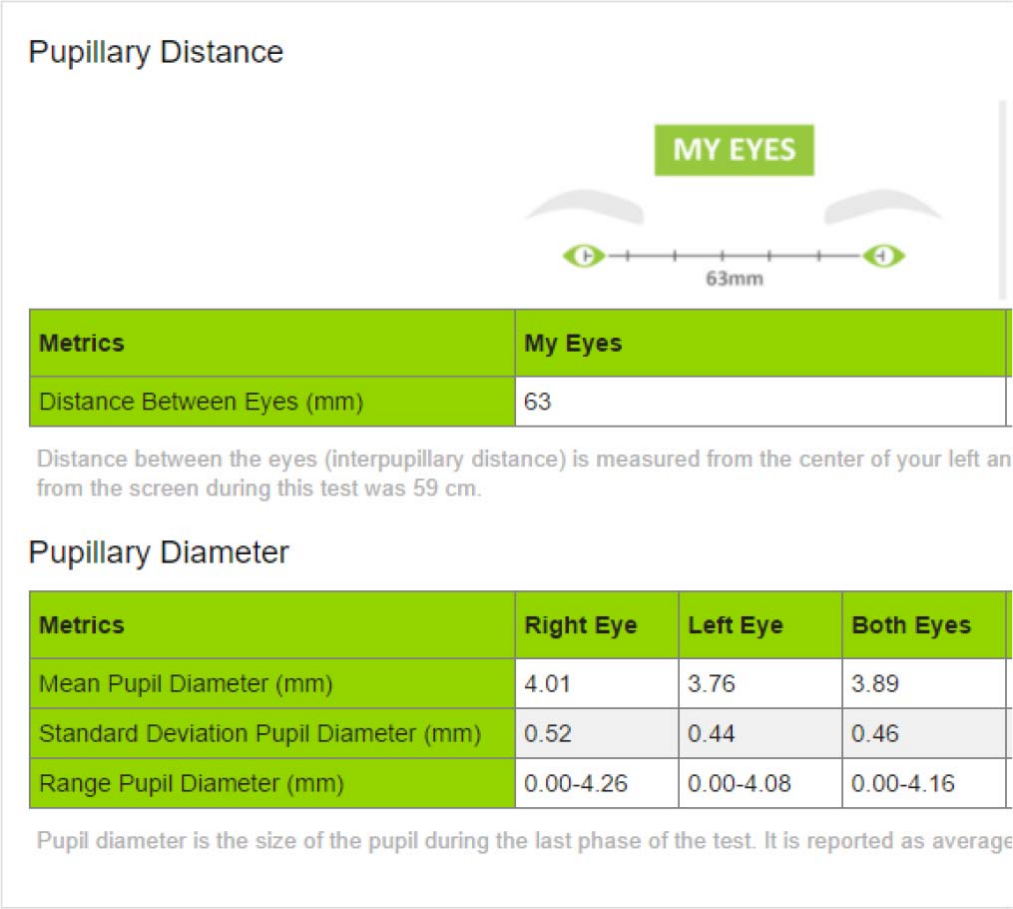
RightEye IPD/PD report.

At the conclusion of the test, a report showed both the IPD and PD results (see Fig. 3).

Participants assigned to the reliability group were then tested a second time using the same procedure as previously described. Time between tests was less than 1 minute.

Participants assigned to the validity group were administered the other clinical tests (RPVS, Essilor Digital CRP, PL850 Pupilometer). For measurement of IPD, the recommended steps outlined in each manual were used.

When using the PL850 and the Essilor Digital CRP, the pupilometer was first turned on. A target distance of 60 cm was set, and measurements were done monocularly. The participant was then instructed to hold the pupilometer up to the eyes the same way one would hold binoculars and to make sure the nose was on the nose rest. The participant was instructed to stare at the red dot through the view windows. Using the left and right eye control buttons, the examiner slid the vertical PD lines over the participant's retinal reflections (the pinpoint of light located inside the pupil). Then once satisfied with each measurement, the “hold” button was pressed to store the readings.

For measurement of PD, participants were requested to sit 60 cm from the computer screen, facing the investigator. The computer screen was not used for the test; however, the luminance from the screen was important to ensure the same conditions as the RightEye IPD/PD test. Computer screen luminance was 85 cd/m^2^ (229 lux), and room luminance with the lights on was 344 cd/m^2^ (102 lux). The investigator then held up the RPVS. The card was held temporal to the participant's eye in the corneal plane. The examiner matched the horizontal PD with the appropriate circle. This procedure was conducted for each eye.

Participants were administered the RPVS, Essilor Digital CRP, PL850 Pupilometer, and RightEye IPD/PD test in random order. Participants were randomly assigned to a group to test either reliability or validity of the RightEye IPD/PD test.

### Validity by Design

Validity by design, also considered face validity or priori validity, determines whether the test measures what is being claimed. The RightEye IPD/PD test has several validity by design elements build into the test. These fall into two categories: (1) test stimuli and (2) testing protocol.

#### Test Stimuli

To obtain accurate IPD, the stimuli must be presented in the center of the screen without deviation from one test to the next. This is obtained through computer programming, allowing the participant to see exactly the same stimuli every time the test is conducted. Furthermore, the initial drop of the stimuli (movement), time, and size reduction of the stimuli encourages the participant to look at the stimuli during the test.

To obtain accurate PD, the luminance level on the screen must remain consistent both during the test and between tests. To ensure this occurs, luminance is preset via software code to prevent any adaptations by a participant or tester.

#### Test Protocol

To ensure accuracy of IPD and PD it is important that three conditions are met: (1) distance from the screen is 60 cm, (2) the eyes remain stationary during the last 700 milliseconds, (3) the participant looks at the stimuli. To assist with these conditions a chin rest is recommended for younger patients or those with certain movement-related disorders. Additionally, error handling is employed using the eye tracker to determine the location of the participants' eyes on the screen, ensuring they are looking at the target during the last 700 milliseconds when IPD and PD are being calculated. If this does not occur, an error message will let the tester know, and the test will be redone. This further enhances the confidence that the participant was confirmed as “on the stimuli” target when the calculations were made.

Hippus is the spasmodic or rhythmic contraction of the pupil of the eye and is an important consideration in both the development of stimulus and the metrics that report the results. Several parameters are in place, in consideration of hippus, to provide accurate results, rather than one point of fluctuation. First, many data samples are collected. The test data are collected 120 times per second, for each eye. Data are collected simultaneously from both eyes. Second, only when the stimulus is stationary and in the center of the screen are data collected and analyzed to minimize hippus volatility that occurs when targets are moving. Third, the testing procedure is standardized; that is, the luminance of the screen as well as stimuli timing and presentation are the same for each participant. Fourth, the resulting normative metrics explore the data from various angles, such as mean, standard deviation, range, variance, kurtosis, and confidence interval (CI; see Table 2). Using these metrics, an individual's results are measured against normative data. Normative data are based on a large sample size of 416 participants. Confidence intervals in the normative data show a very small range between the 95% CI upper and lower bounds. All these factors combined are considered in response to hippus and therefore allow the results of an individual's data to be referenced in confidence to the pool of data reported while considering the hippus effect.

**Table 1:**
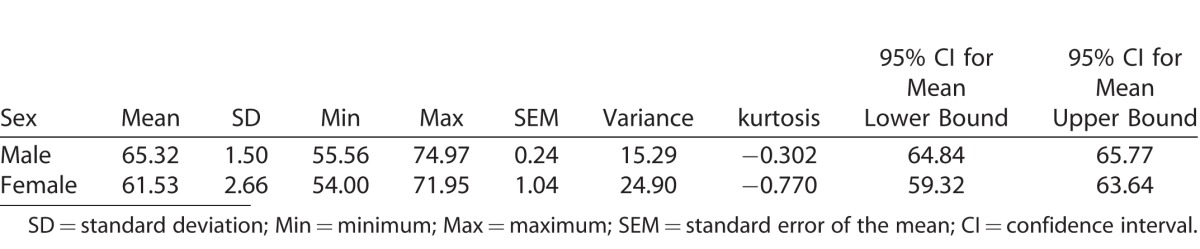
Normative Data for RightEye IPD

**Table 2:**
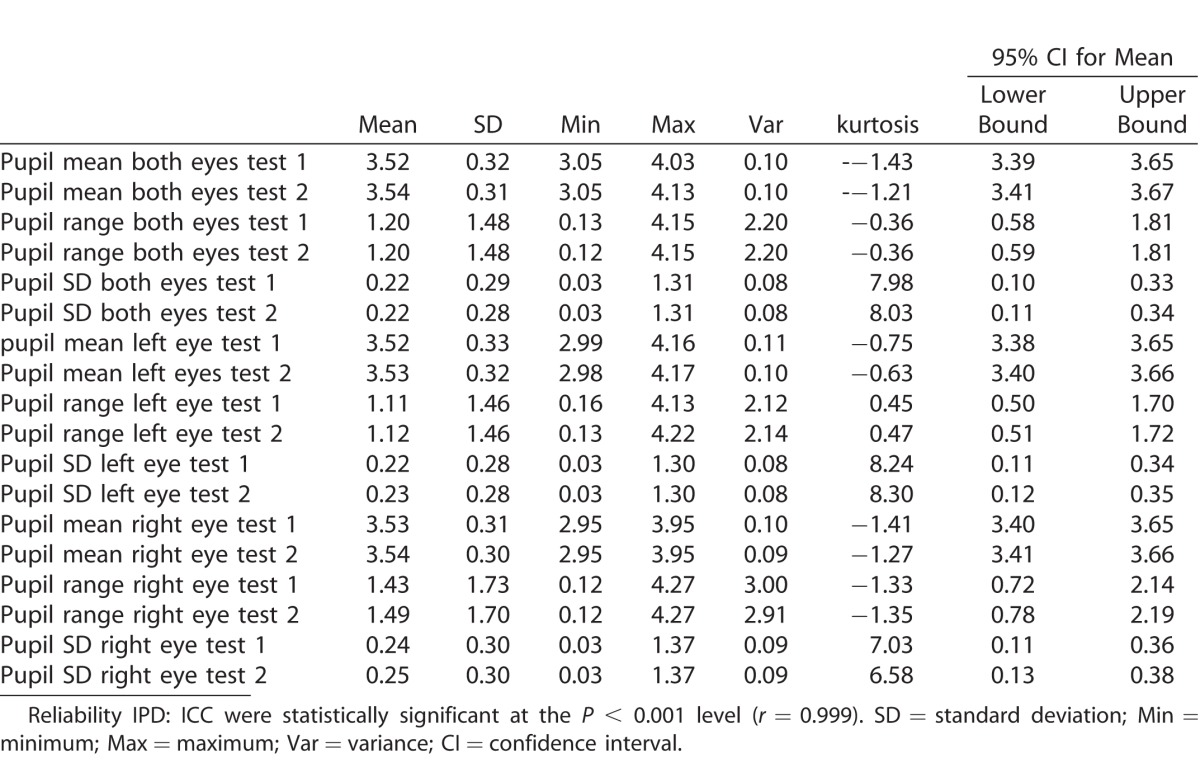
Normative Data for RightEye PD

Furthermore, to ensure overall testing accuracy, each tester was trained on how to run each test with accuracy and consistency and was given 1 hour of dedicated training. This was concluded with a test in the form of a demonstration to an experienced tester, requiring a “passing” grade prior to testing any participants.

### Data Analysis

#### Reliability

Test-retest reliability for IPD and PD was analyzed with intraclass correlation coefficients (ICCs) between trials with Cronbach's alpha (CA) and the standard error of mean (SEM) for each ICC. Alpha level was set at *P* < 0.05 for all statistical tests. The ICC indicates the relative reliability and is interpreted using the following criteria: ICC > 0.75 specifies excellent reliability and 0.40 < ICC > 0.74 represents fair to good reliability.

#### Validity

Convergent validity was investigated by calculating the bivariate correlation coefficient of the RightEye IPD/PD test and PL850 Pupilometer and the bivariate correlation coefficient of the RightEye IPD/PD test and Essilor Digital CRP. Validity was also explored by examining the convergent findings within the analysis of variance test of each measure (RightEye IPD/PD test, PL850 Pupilometer, Essilor Digital CRP) and by designing the test to meet with current standard measure of IPD and PD. Raw data for each test were collected as a whole number value; however, the analysis is reported as mean values carried out to two decimal points.

## Results

Normative data for IPD is found in Table 1. In addition, Table 2 demonstrates the normative data for PD. Following CA, ICCs and associated SEM for test-to-test reliability (test 1 and test 2) are reported for IPD and PD. Observations for all variables demonstrated strong reliability.

Observations for IPD demonstrated perfect reliability with CA = 1.00, which is well above the acceptable level of 0.70 and ICC = 1. Calculated SEMs suggest the measures are capable of accurate assessment of IPD (test 1: SEM = 0.148; test 2: SEM = 0.440). Age had no effect on reliability for IPD.

### 

#### Reliability PD

Cronbach's alpha, ICC, and associated SEM for test-to-test reliability (test 1 and test 2) are reported in Table 3. Observations for all variables demonstrated strong reliability. All CAs are above the acceptable level of 0.7, which is considered ideal (Tavakol and Dennick).^30^ Calculated SEMs represent an accurate assessment of PD. In addition, all ICCs were statistically significant at the *P* < 0.001 level. There was no effect of age on reliability for PD.

**Table 3:**
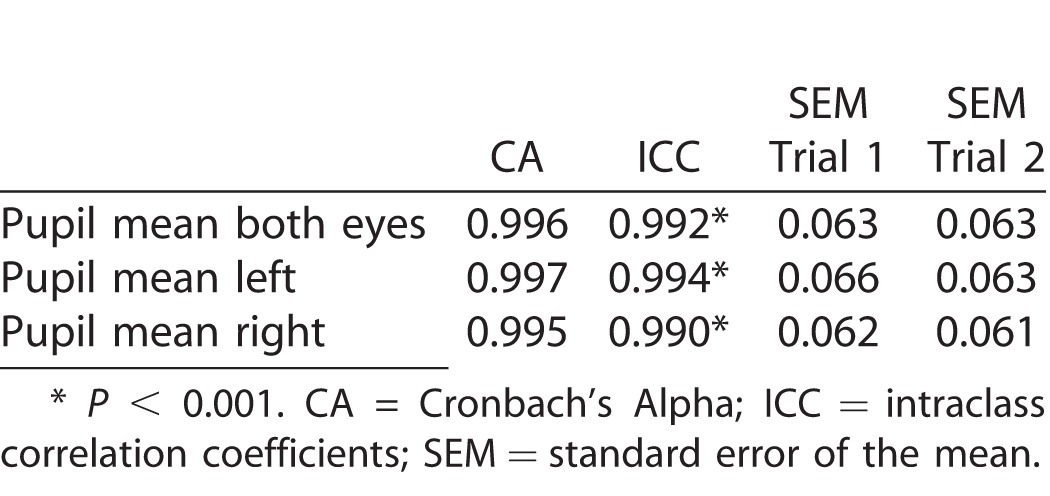
PD Reliability

#### Validity IPD

**Figure 4:**
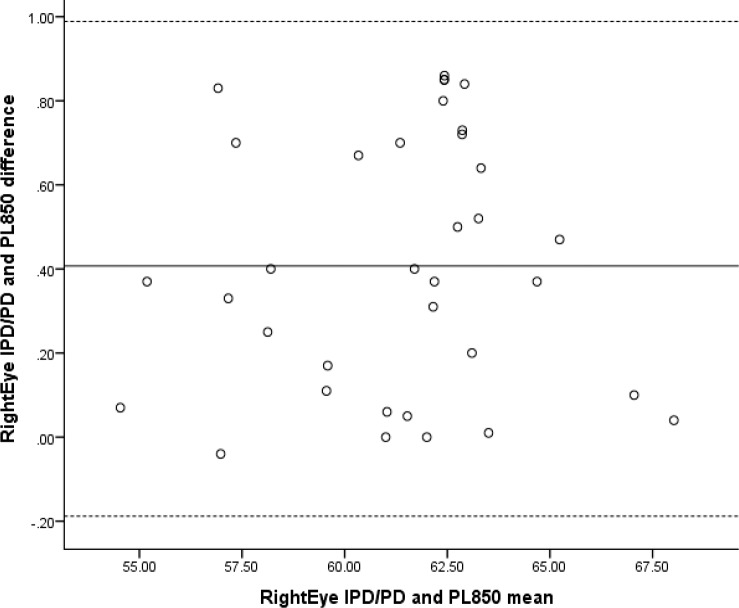
Bland-Altman plot comparing RightEye IPD/PD and PL850 Pupilometer. (Note: for all Bland-Altman plots, the solid line shows the mean difference, whereas the dashed line shows 95% limits of agreement).

Correlation analyses revealed high positive significant correlations between the RightEye IPD/PD test and PL850 Pupilometer (*r* = 0.971, *n* = 659, *P* < 0.001) and Essilor Digital CRP (*r* = 0.985, *n* = 659, *P* < 0.001). The Bland-Altman plots for IPD and PL850 (see Fig. 4) demonstrated no proportional bias and a mean difference of 0.407 (95% CI: 0.3026–0.5117).

**Figure 5:**
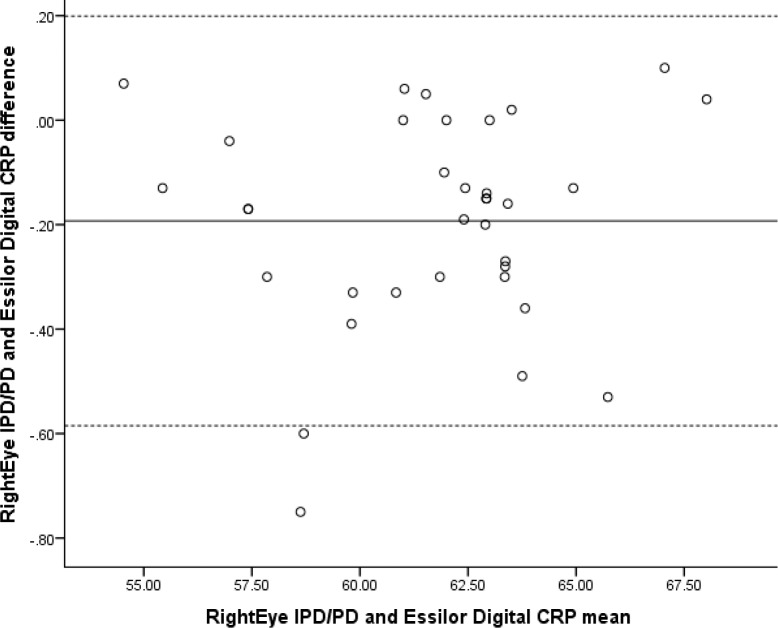
Bland-Altman plot comparing Righteye IPD/PD and Essilor Digital CRP.

Similarly, the Bland-Altman plots for IPD and Essilor Digital CRP demonstrate no proportional bias (see Fig. 5).

Two linear regression analyses were conducted: one to evaluate the proportional bias of RightEye IPD/PD test and PL850 and one to evaluate the proportional bias of RightEye IPD/PD test and the Essilor Digital CRP. Both regression analyses demonstrated a nonsignificant finding (*R*^2^= 0.056, *F*[1, 49] = 0.105, *P* = 0.748, and *R*^2^= 0.095; *F*[1, 49] = 0.301, *P* = 0.587, respectively).

Validity was further demonstrated in group differences analysis as all three variables (RightEye IPD/PD test, PL850 Pupilometer, and Essilor Digital CRP) produced equivalent results (see Table 4). In addition, validity was also established by design (see Methods section).

**Table 4:**
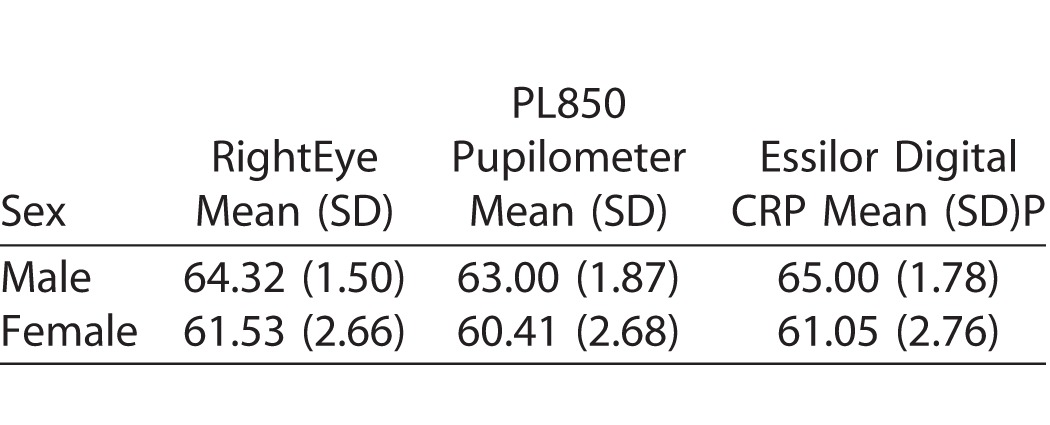
Descriptive Statistics for IPD

#### Validity PD

**Figure 6:**
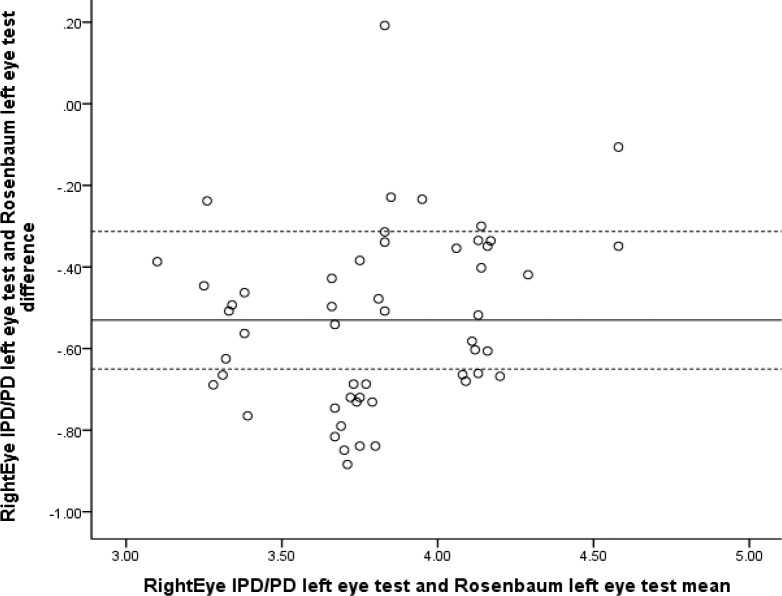
Bland-Altman plot comparing RightEye IPD/PD left eye test and Rosenbaum left eye test.

**Figure 7:**
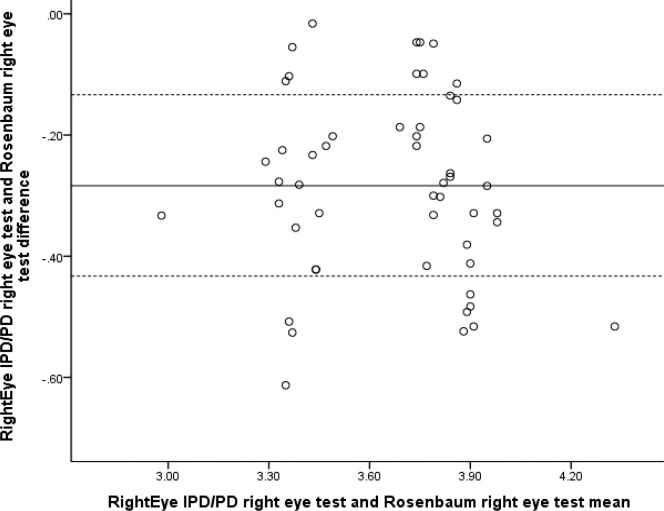
Bland-Altman plot comparing RightEye PD right eye test and Rosenbaum right eye test.

Correlation analyses for PD validity demonstrated high positive significant correlation between the RightEye IPD/PD left eye test and Rosenbaum left eye test (*r* = 0.883, *n* = 52, *P* < 0.001) and high significant correlation between the RightEye PD right eye test and Rosenbaum right eye test (*r* = 0.851, *n* = 52, *P* < 0.001). Rosenbaum method showed higher average PDs compared to the RightEye IPD/PD test (see Table 5); however, Bland-Altman plots (see Fig. 6 and Fig. 7) and regression analysis indicated no proportional bias for both left and right eye comparisons of the RightEye test and the Rosenbaum test (*R*^2^= 0.238, *F*[1, 49] = 0.299, *P* = 0.09, and *R*^2^= 0.110; *F*[1, 49] = 0.607, *P* = 0.439, respectively). ). In addition, validity was also established by design (see Methods section).

**Table 5:**
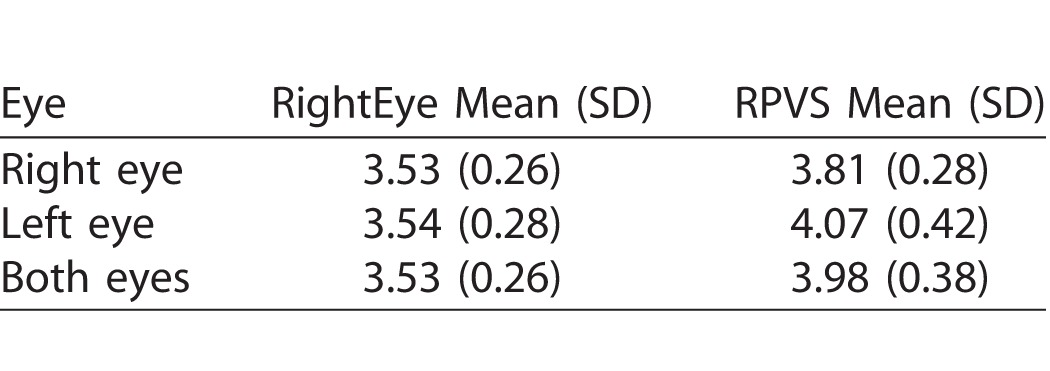
Descriptive Statistics for PD

## Discussion

The purpose of this study was to determine the reliability of IPD and PD measures using an infrared eye tracker (SMI 12” 120 Hz) and central point stimuli (designed by RightEye, LLC). Results reveal the test-to-test reliability is strong for both IPD and PD. For all metrics, CAs are above the acceptable level of 0.7, which is considered ideal.^30^

Past research by Obstfeld and Chou^12^ found consistent readings against nine of the leading pupilometers within the limits of clinically acceptable parameters (that is, mean differences of 2.3 mm and SD = 0.26 mm). Results from this study are at least in line with, if not stronger than, the pupilometers as the correlation (CA) for IPD is 1.0 and for PD is 0.995–0.997.

Although pupilometers have been examined and been found to be reliable,^12^ measures for IPD, consistent measurement of PD using various tools (lens systems, infrared light, and wavelength aberrometers) has been more difficult to demonstrate. In fact, Lawrence et al.,^31^ after testing PD in 200 healthy eyes, concluded that “due to the complex interaction among observer, pupillometry technique, and iris color, one cannot compare the four techniques to each other with the same observer, nor can one compare the two observers irrespective of the technique.” Past research has also found poor reliability in pupilometers due to examiner error such as incorrect positioning.^5,13^ The results of this study, however, indicate that the test is consistent over time given the same environmental conditions while following the testing procedure outlined. There was also no effect of age on reliability for IPD or PD. Consistency in output is important but does not demonstrate accuracy. Therefore, our second objective was to determine if the RightEye IPD/PD test is accurate across all measures.

The RightEye IPD test compared to the PL850 Pupilometer and the Essilor Digital CRP determined IPD to be accurate and valid. Results revealed significant positive correlations (*P* < 0.001) when comparing output of the RightEye IPD results compared to both the PL850 Pupilometer and the Essilor Digital CRP, therefore suggesting that the RightEye IPD test is, at a minimum, as accurate as these other measures. Descriptive statistics (means and standard deviations) across all measures fall within a similar range, with the PL850 producing lower IPDs than the RightEye and Essilor Digital CRP tests.

The validity of the RightEye PD test compared to the RPVS for PD measures was also examined and resulted in a high positive correlation between the two tools (*P* < 0.001). Past research has found that the RPVS overestimated PD and may not be appropriate for evaluating PD for refractive surgery.^16^ In this study, the RPVS also showed a higher average PD than the RightEye PD test.

Accurate and reliable measures of PD can be difficult for various reasons (hippus, eye color, luminance, and technique used). Nevertheless, it is important in modern ophthalmologic practice because of modern refractive surgery.^32^ Schmidt et al.,^14^ found statistically significant deviations in lens systems, infrared light, and wavelength aberrometers. Various infrared eye trackers have found that these eye trackers can over- and underestimate PD depending on eye position.^18^ When the eye is not positioned straight ahead, the pupil may appeared squashed, giving inaccurate readings. Therefore, the design of this experiment included stimuli that measure PD at a central point of fixation. Results revealed central PD measures ranged from 0.26 to 0.28 mm. These results are improved compared with past eye-tracking research,^18^ showing that central PD measures ranged from 0.3 to 1 mm. This may be due to the location of the stimuli when measuring PD with eye tracking.

The third objective of this study was to examine the data to establish normative references, for which individuals can measure themselves. Normative data are based on large sample size of 416 participants that were well matched in terms of gender (45% males and 55% females) across a wide range of age (18–73 years: M = 42, SD = 8.7) and ethnic backgrounds. These data are an adequate reference for comparison for people with normal vision development.

This experiment is not without limitations. Future studies should measure the RightEye PD test output against more sophisticated methods of measurement such as monocular autorefractors (e.g., Marco Nidek ARK-530A, Keeler PupilScan II, or NeurOptics PLR200). Future research should also examine the RightEye stimuli against other eye trackers (e.g., Tobii, SR Research and EyeTribe).

These results are limited to participants with normal visual function. Additional experimentation should consider those with refractive errors, nystagmus, and other vision-related disorders to determine the tests' reliability.

A further exploration into various demographic factors such as ethnicity, gender, and age is needed. Past research has found differences across these variables that, with more specific analysis, would allow people to measure themselves against a more similar normative comparison.
